# The minor C-allele of rs2014355 in *ACADS *is associated with reduced insulin release following an oral glucose load

**DOI:** 10.1186/1471-2350-12-4

**Published:** 2011-01-06

**Authors:** Malene Hornbak, Karina Banasik, Johanne M Justesen, Nikolaj T Krarup, Camilla H Sandholt, Åsa Andersson, Annelli Sandbæk, Torsten Lauritzen, Charlotta Pisinger, Daniel R Witte, Thorkild IA Sørensen, Oluf Pedersen, Torben Hansen

**Affiliations:** 1 Marie Krogh Center for Metabolic Research, Section of Metabolic Genetics, Faculty of Health Sciences, University of Copenhagen, Denmark; 2 Faculty of Pharmaceutical Sciences, University of Copenhagen, Copenhagen, Denmark; 3 Hagedorn Research Institute, Gentofte, Denmark; 4 Institute of Biomedical Science, Faculty of Health Sciences, University of Copenhagen, Copenhagen, Denmark; 5 Novo Nordisk A/S, Medical and Science, Development Projects, Bagsværd, Denmark; 6 Department of General Practice, University of Aarhus, Aarhus, Denmark; 7 Research Centre for Prevention and Health, Glostrup University Hospital, Glostrup, Denmark; 8 Steno Diabetes Center, Gentofte, Denmark; 9 Institute of Preventive Medicine, Copenhagen University Hospital, Center for Health and Society, Copenhagen, Denmark; 10 Faculty of Health Sciences, University of Aarhus, Aarhus, Denmark; 11 Faculty of Health Sciences, University of Southern Denmark, Odense, Denmark

## Abstract

**Background:**

A genome-wide association study (GWAS) using metabolite concentrations as proxies for enzymatic activity, suggested that two variants: rs2014355 in the gene encoding short-chain acyl-coenzyme A dehydrogenase (*ACADS*) and rs11161510 in the gene encoding medium-chain acyl-coenzyme A dehydrogenase (*ACADM*) impair fatty acid β-oxidation. Chronic exposure to fatty acids due to an impaired β-oxidation may down-regulate the glucose-stimulated insulin release and result in an increased risk of type 2 diabetes (T2D). We aimed to investigate whether the two variants associate with altered insulin release following an oral glucose load or with T2D.

**Methods:**

The variants were genotyped using KASPar^® ^PCR SNP genotyping system and investigated for associations with estimates of insulin release and insulin sensitivity following an oral glucose tolerance test (OGTT) in a random sample of middle-aged Danish individuals (*n*_*ACADS *_= 4,324; *n*_*ACADM *_= 4,337). The T2D-case-control study involved a total of ~8,300 Danish individuals (*n*_*ACADS *_= 8,313; *n*_*ACADM *_= 8,344).

**Results:**

In glucose-tolerant individuals the minor C-allele of rs2014355 of *ACADS *associated with reduced measures of serum insulin at 30 min following an oral glucose load (per allele effect (β) = -3.8% (-6.3%;-1.3%), *P *= 0.003), reduced incremental area under the insulin curve (β = -3.6% (-6.3%;-0.9%), *P *= 0.009), reduced acute insulin response (β = -2.2% (-4.2%;0.2%), *P *= 0.03), and with increased insulin sensitivity ISI_Matsuda _(β = 2.9% (0.5%;5.2%), *P *= 0.02). The C-allele did not associate with two other measures of insulin sensitivity or with a derived disposition index. The C-allele was not associated with T2D in the case-control analysis (OR 1.07, 95% CI 0.96-1.18, *P *= 0.21). rs11161510 of *ACADM *did not associate with any indices of glucose-stimulated insulin release or with T2D.

**Conclusions:**

In glucose-tolerant individuals the minor C-allele of rs2014355 of *ACADS *was associated with reduced measures of glucose-stimulated insulin release during an OGTT, a finding which in part may be mediated through an impaired β-oxidation of fatty acids.

## Background

Acute exposure of free fatty acids (FFA) to the pancreatic β-cells is known to both stimulate insulin secretion and modulate the glucose-stimulated insulin secretion (GSIS) whereas chronic exposure leads to desensitization of the insulin receptors and decreased function of the pancreatic β-cells. Also, chronic exposure to FFA is associated with insulin resistance and increased risk of developing diabetes mellitus [1-9].

Recently, Gieger *et al*. (2008) conducted a genome-wide association study (GWAS) in 284 healthy men characterized by quantitative measurements of 363 metabolites obtained from fasting serum samples, and used concentration ratios for the metabolites as proxies for enzymatic activity [10]. They identified with genome-wide significance variants in two genes, one encoding short-chain acyl-coenzyme A dehydrogenase (*ACADS) *which is expressed in mitochondria and target short chain fatty acid (SCFA), and one encoding medium-chain acyl-coenzyme A dehydrogenase *(ACADM) *that target medium chain C4-C12 fatty acids (MCFA), where the corresponding metabolic phenotype matched the biochemical pathway of the enzymes. A strong association (*P *= 9.3 × 10^-17^) was found between rs2014355 in *ACADS *and the ratio between the short-chain acylcarnitines C3 and C4. Similarly, they found a strong association (*P *= 7.6 × 10^-17^) between rs11161510 in *ACADM *and the ratio between medium-chain acylcarnitines C8 and C12 [10]. These findings indicate that individuals homozygous for the minor alleles of the variants in *ACADS *and *ACADM *have reduced dehydrogenase activity and therefore are likely to show impaired β-oxidation. For instance, in situations of prolonged starvation, these individuals may become hypoglycemic and show hypoglycemia-related symptoms [10].

In pancreatic β-cells, glucose is the primary source of fuel to stimulate insulin secretion [1,2,9]. However, when the glucose concentration in plasma is low, fatty acids (FA) are produced by *de novo *lipolysis of endogenous triglycerides (TG) increasing the level of cytoplasmic long-chain Coenzyme A molecules. Subsequently, these molecules are transported into the mitochondria (via carnitine-palmitoyl-transferase I (CPT-I)) for β-oxidation leading to increased cellular ATP levels. Thus, the cell can use FAs as fuels to produce both energy and stimulate the insulin secretion [8]. Conversely, when plasma glucose concentration is elevated, β-oxidation of FAs is inhibited due to allosteric inhibition of CPT-I from malonyl-CoA with a subsequent increase in FFA esterification and synthesis of complex lipid molecules such as diacylglycerol, triacylglycerol and fatty acids [1,7,8,11,12]. Accordingly, during chronically elevated circulating FFA levels it is hypothesized that the synergism of both glucose- and lipid-stimulated insulin-secretion elevates the basal insulin secretion causing hyperinsulinemia which may eventually exhaust the β-cell and desensitize the insulin receptors [5]. Chronic FA exposure may thus down-regulate the GSIS with overt diabetes as a consequence [5].

*ACADS *and *ACADM *encode Acyl-CoA dehydrogenases, which are enzymes that catalyze the initial step in the β-oxidation of FAs in the mitochondria, each targeting FAs of varying length. In humans, deficiency of these enzymes leads to low tissue levels of dehydrogenase activity and improper break-down of SCFA and MCFA. Consequently, the FAs cannot be converted into energy in situations where the glucose-concentration is low resulting in hypoglycemia, lethargy, hypotonia, and seizures [13-15]. *ACADS *is very abundant in the pancreatic β-cell, indicating that it plays an important regulatory function, and decreased activity of short-chain dehydrogenases in the pancreatic β-cell is hypothesized to impair insulin secretion [15]. Studies in mice deficient for ACADS and ACADM showed that the mice developed fatty liver, changes in hepatic carbohydrate metabolism, and hypoglycemia when fasted, and studies by Herrema *et al. *indicated that peripheral rather than hepatic consequences might underlie the hypoglycemia associated with disorders of mitochondrial fatty acid oxidation [16-19].

The aim of the present study was to investigate rs2014355 of *ACADS *and rs11161510 of *ACADM *in relation to indices of insulin release, insulin sensitivity, and fasting serum lipid levels in a large random sample of middle-aged individuals (*n *= 6,162); also we examined the putative relation to T2D prevalence in a case-control study involving ~8,300 Danish individuals (*n*_*ACADS *_= 8,313; *n*_*ACADM *_= 8,344).

## Methods

### Study population

The study population included 10,276 Danes from four different study groups: 1) The Inter99 cohort which is a randomized, non-pharmacological intervention study for the prevention of ischemic heart disease in people recruited as a random sample and conducted at the research Centre for Prevention and Health in Glostrup, Copenhagen (*n *= 6,162) (ClinicalTrials.gov ID-no: NCT00289237); 2) T2D patients sampled through the out-patient clinic at Steno Diabetes Center (SDC) (*n *= 1,695); 3) a randomly recruited sample of middle-aged glucose-tolerant participants examined at SDC (*n *= 810); and 4) T2D patients from the Addition Denmark screening study cohort (Anglo-Danish-Dutch Study of Intensive Treatment in People with Screen-Detected Diabetes in Primary Care) (ClinicalTrials.gov ID-no: NCT00237548), which is a population-based, high-risk screening and intervention study for T2D in general practice (*n *= 1,609) [20,21]. Characteristics for individuals included in the study are shown in Additional file 1. All participants in study group 1 and 3 underwent a standard 75 g oral glucose tolerance test (OGTT). T2D and glucose tolerance status were diagnosed according to the World Health Organization 1999 criteria [22].

Analyses of quantitative diabetes-related traits were performed in glucose-tolerant individuals from the Inter99 cohort (*n*_*ACADS *_= 4,324; *n*_*ACADM *_= 4,337). Analyses of fasting serum lipid levels were performed in glucose-tolerant and treatment-naïve individuals from the Inter99 cohort (*n*_*ACADS *_= 5,702; *n*_*ACADM *_= 5,731). Study group 3 was excluded from the quantitative trait analyses due to missing phenotypes. All T2D patients and glucose-tolerant individuals were included in the case-control study of T2D (*n*_*ACADS *_= 8,313; *n*_*ACADM *_= 8,344). Participants were of Danish nationality and informed written consent was obtained from all individuals before participation. The studies were conducted in accordance with the Declaration of Helsinki II and were approved by the local Ethical Committees of Copenhagen and Aarhus.

### Biochemical and anthropometric measures

Height (without shoes) and weight were measured in light indoor clothing, and BMI was calculated as weight (kg)/height^2 ^(m^2^). Waist circumference was measured in the upright position midway between the iliac crest and the lower costal margin, and hip circumference was measured at its maximum [21]. Blood samples were drawn after a 12 h overnight fast. Plasma glucose was analyzed by a glucose oxidase method (Granutest; Merck, Darmstadt, Germany) and serum insulin (excluding des-31,32 and intact proinsulin) was measured using the Autodelfia insulin kit (Perkin-Elmer/Wallac, Turku, Finland). Serum triacylglycerol was analyzed using enzymatic colorimetric methods (GPO-PAP and CHOD-PAP; Roche Molecular Biochemicals, Mannheim, Germany). Serum C-peptide concentrations were measured by a time-resolved fluoroimmunoassay (AutoDELFIA C-peptide kit; Perkin-Elmer/Wallac, Turku, Finland). Homeostasis model assessment of insulin resistance (HOMA-IR) was calculated as: (fasting plasma glucose (mmol/l) × fasting serum insulin (pmol/l))/22.5 [23]. BIGTT-insulin sensitivity index (BIGTT-S_i_) and BIGTT-acute insulin response (BIGTT-AIR) use information on sex and BMI combined with analysis of plasma glucose and serum insulin levels at time points 0, 30, and 120 min to provide indices for S_I _and AIR that highly correlate with indices obtained during an intravenous glucose tolerance test. These indices were calculated as described elsewhere [24]. β-cell function was further estimated as the insulinogenic index, calculated as: (serum insulin at 30 min (pmol/l) - fasting serum insulin (pmol/l))/(plasma glucose at 30 min (mmol/l) - fasting plasma glucose (mmol/l)). A disposition index was calculated as insulinogenic index/HOMA-IR. Matsuda whole body insulin sensitivity index (ISI_Matsuda_) was calculated as (10,000/√ (fasting plasma glucose × fasting serum insulin) × (mean plasma glucose × mean serum insulin during OGTT)) [25]. Area under the curve (AUC) for plasma glucose, serum insulin and serum C-peptide were calculated using the trapezoidal method. The AUC for insulin/AUC for glucose ratio was calculated as AUC for insulin/AUC for glucose.

### Genotyping

Genotyping was performed using KASPar^® ^PCR SNP genotyping system (KBiosciences, Herts, UK) with a success rate >97%. Discordance was <0.1% as judged from re-genotyping of 1187 random duplicate samples. Allele frequencies were in accordance with HapMap (CEU population) and the study by Gieger *et al.*, and obeyed Hardy-Weinberg equilibrium (*P *> 0.05) [10].

### Statistical analysis

A general linear model was applied for testing quantitative traits in relation to genotype. Non-normally distributed data (measures of serum insulin, serum triglyceride, and serum C-peptide levels, HOMA-IR, insulinogenic index, AUC for insulin/AUC for glucose ratio, ISI_Matsuda_, and BIGTT-AIR) were logarithmically transformed before analyses. Logistic regression was used to examine differences in genotype distribution in the case-control studies. All studies were performed in R 2.10.0 assuming an additive model and adjusted for age, sex, and BMI. In a subset of the analyses, estimates of β-cell function were additionally adjusted for fasting serum lipid levels (cholesterol or triglyceride). *P *< 0.05 were considered significant. The statistical power calculations were done using CaTS, power calculations for large genetic association studies, available at http://www.sph.umich.edu/csg/abecasis/cats/. The statistical power to detect an OR of 1.10 was estimated to be 80% for rs2014355 of *ACADS *and 86% for rs11161510 of *ACADM*, respectively. (*P *< 0.05, MAF = 24%, T2D risk = 0.08).

## Results

### Association with T2D-related quantitative traits

The impact of *ACADS *rs2014355 and *ACADM *rs11161510 on OGTT-derived diabetes-related quantitative traits was evaluated in a random sample of glucose-tolerant individuals from the Inter99 cohort (Table 1 and 2). Assuming an additive genetic model, carriers of the minor C-allele of *ACADS *rs2014355 had decreased measures of serum insulin (per allele effect (β) = -3.8% (-6.3%;-1.3%), *P *= 0.003) at 30 min, and decreased incremental AUC for serum insulin from 0-120 minutes during an OGTT (β = -3.6% (-6.3%;-0.9%), *P *= 0.009) (Table 1). Also, measures of serum C-peptide at 30 min (β = -1.8% (-3.4%;-0.1%), *P *= 0.03), and incremental AUC for serum C-peptide from 0-120 minutes (β = -1.8% (-3.4%;-0.1%), *P *= 0.03) during an OGTT were decreased for carriers of the rs2014355 C-allele (Table 1). Additionally, the rs2014355 C-allele of *ACADS *associated with reduced β-cell response measured as BIGTT-AIR (β = -2.2% (-4.2%;0.2%), *P *= 0.03) and the AUC for insulin/AUC for glucose ratio (β = -0.03%(-0.05%;-0.01%), *P *= 0.01). The C-allele did not associate with BIGTT-S_i _or HOMA-IR or with a HOMA-IR-derived disposition index; however, the variant associated with increased insulin sensitivity according to ISI_Matsuda _(β = 2.9% (0.5%;5.2%), *P *= 0.02) (Table 1). When measures of insulin release were adjusted for either fasting levels of serum cholesterol or triglyceride, the effects were unchanged (data not shown). However, adjusting ISI_Matsuda _for serum cholesterol or triglyceride levels strengthened the association with whole body insulin sensitivity (*P *= 0.01, data not shown). Further, when investigating whether rs2014355 influences lipid characteristics in normoglycemic and hyperglycemic individuals, respectively, no effect was evident among normoglycemic individuals (Table 2). However, hyperglycemic (IFG, IGT, and screen-detected T2D) individuals showed a significant genotype-dependent decrease in fasting serum HDL-cholesterol levels (β = -0.036 (-0.067; -0.005), *P *= 0.02) and increased fasting serum triglyceride levels (β = 5.2% (0.4%; 10.0%), *P *= 0.02) among homozygous minor C-allele carriers (Table 2). No significant associations were observed in relation to levels of plasma glucose for this variant (Table 1).

**Table 1:** Anthropometric and metabolic characteristics of successfully genotyped glucose-tolerant Danes from the Inter99 cohort stratified according to ACADS rs2014355 (T/C) and ACADM rs11161510 (C/T) genotype, respectively.

		WT	HE	HO	β (95% CI)	***P***_**add**_		
*n *(men/women)	*ACADS*	2,576 (1,208/1,368)	1,496 (694/802)	252 (105/147)				
	*ACADM*	2,121(991/1,130)	1,821(825/996)	395(186/209)				

Age (years)		45 ± 8	45 ± 8	45 ± 8				
		45 ± 8	45 ± 8	46 ± 8				

BMI (kg/m^2^)		25.5 ± 4.1	25.4 ± 3.9	25.8 ± 4.0	0.03 (-0.16;0.23)	0.74		
		25.5 ± 4.0	25.6 ± 4.2	25.4 ± 3.8	0.03 (-0.16;0.21)	0.79		

**Plasma glucose (mmol/l)**								

Fasting	*ACADS*	5.3 ± 0.4	5.3 ± 0.4	5.3 ± 0.4	0.005 (-0.013;0.024)	0.55		
	*ACADM*	5.3 ± 0.4	5.3 ± 0.4	5.3 ± 0.4	0.016 (-0.001;0.033)	0.06		

30-min during		8.2 ± 1.5	8.2 ± 1.5	8.1 ± 1.6	-0.05 (-0.12;0.02)	0.18		
an OGTT		8.2 ± 1.5	8.1 ± 1.5	8.3 ± 1.5	-0.02 (-0.08;0.05)	0.60		

120-min during		5.5 ± 1.1	5.5 ± 1.2	5.5 ± 1.1	-0.03 (-0.08;0.03)	0.36		
an OGTT		5.5 ± 1.1	5.5 ± 1.1	5.5 ± 1.2	-0.004 (-0.05;0.05)	0.88		

Incremental		183.7 ± 100.3	177.9 ± 102.0	175.5 ± 100.7	-4.90 (-9.82;0.01)	0.05		
AUC		183.7 ± 99.3	177.5 ± 100.8	183.3 ± 103.7	-2.9 (-7.5;1.6)	0.21		

**Serum insulin (pmol/l)**								

Fasting	*ACADS*	31 (22-46)	31 (22-46)	29 (23-41)	-1,9% (-4.3%;0.6%)	0.14		
	*ACADM*	31 (22-46)	31 (22-45)	31 (23-46)	0.3% (-2.0%;2.6%)	0.83		

30-min during		246 (177-356)	239 (175-329)	234 (181-302)	-3.8% (-6.3%;-1.3%)	0.003		
an OGTT		239 (175-337)	244 (178-351)	245 (173-348)	0.5% (-1.8%;2.8%)	0.65		

120-min during		137 (88-212)	139 (86-209)	130 (87-189)	-1.1% (-4.5%;2.4%)	0.51		
an OGTT		140 (87-212)	136 (88-208)	129 (80-214)	-2.8% (-5.9%;0.3%)	0.08		

Incremental		18,220 (12,600-25,830)	17,260 (12,700-23,980)	16,200 (12,630-22,720)	-3.6% (-6.3%;-0.9%)	0.009		
AUC		17,740 (12,720-24,780)	17,780 (12,600-25,380)	17,670 (12,490-25,790)	0.1% (-2.3%;2.6%)	0.91		

**Serum C-peptide (pmol/l)**								

Fasting	*ACADS*	495 (392-632)	499 (394-642)	484 (399-627)	0.2% (-1.3%;1.8%)	0.78		
	*ACADM*	498 (393-639)	494 (392-632)	496 (405-627)	-0.4% (-1.8%;1.0%)	0.61		

30-min during		1,910 (1,490-2,390)	1,840 (1,470-2,310)	1,820 (1,450-2,268)	-1.8% (-3.4%;-0.1%)	0.03		
an OGTT		1,880 (1,480-2,350)	1,870 (1,490-2,398)	1,870 (1,450-2,295)	0.3% (-1.2%;1.8%)	0.71		

120-min during		1,960 (1,500-2,490)	1,980 (1,490-2,490)	1,940 (1,450-2,410)	-0.2% (-2.0%;1.7%)	0.84		
an OGTT		1,980 (1,500-2,508)	1,960 (1,490-2,480)	1,960 (1,480-2,490)	-1.3% (-3.0%;0.4%)	0.13		

Incremental		149,800 (117,600-185,000)	147,300 (117,700-181,600)	138,500 (117,400-178,400)	-1.8% (-3.4%;-0.1%)	0.03		
AUC		148,800 (117,100-184,100)	148,000 (118,500-183,200)	147,900 (116,600-183,800)	-0.1% (.1.7%;1.4%)	0.85		

**Derived indices**								

HOMA-IR	*ACADS*	7.5 (5.2-11.0)	7.4 (5.1-11.0)	6.8 (5.3-9.6)	-1.8% (-4.4%;0.7%)	0.16		
(mmol/l*pmol/l)	*ACADM*	7.4 (5.1-11.0)	7.5 (5.2-10.9)	7.4 (5.3-11.0)	0.6% (-1.8%;3.0%)	0.63		

BIGTT-S_i_		10.3 ± 3.7	10.4 ± 3.8	10.6 ± 3.5	0.14 (-0.05;0.34)	0.14		
		10.4 ± 3.7	10.3 ± 3.7	10.3 ± 3.6	-0.01 (-0.19;0.17)	0.93		

Insulinogenic		79.9 (48.9-130.7)	74.7 (49.2-123.4)	77.1 (51.5-121.7)	-0.8% (4.2%;2.5%)	0.63		
index		75.9 (48.2-123.9)	80.0 (51.1-136.8)	75.8 (46.5-126.4)	2.6% (0.5%;5.7%)	0.10		

Disposition		9.3 (5.1-17.7)	9.3 (5.1-18.1)	10.6 (5.7-18.7)	0.7% (-3.1%;4.5%)	0.73		
index		9.4 (5.3-18.2)	9.4 (5.0-17.6)	9.2 (4.9-16.7)	-3.5% (7.9%;0.8%)	0.11		

ISI_Matsuda_		25.2 (17.8-35.4)	26.1 (18.2-35.9)	27.9 (20.1-36.0)	2.9% (0.5%;5.2%)	0.02		
		26.0 (18.0-35.4)	25.3 (17.9-35.7)	25.6 (18.1-35.7)	-0.2% (2.3%;2.0%)	0.87		

BIGTT-AIR		1,690 (1,344-2,149)	1,655 (1,356-2,056)	1,625 (1,317-2,067)	-2.2% (-4.2%;0.2%)	0.03		
		1,668 (1,356-2,099)	1,684 (1,351-2,140)	1,712 (1,303-2,048)	0.2% (-1.6%;2.0%)	0.83		

AUCinsulin/		27.8 (20.2-38.4)	27.0 (20.5-36.1)	24.9 (19.8-35.1)	-0.03%(-0.05%;-0.01%)	0.01		
AUCglucose		27.1 (20.3-37.0)	27.6 (20.4-38.2)	27.7 (19.8-38.3)	0.002%(-0.019%; 0.023%)	0.87		

**Genotype distribution and allele frequency among patients with T2D and glucose-tolerant control individuals**								
		*n (men/women)*	WT (%)	HE (%)	HO (%)	MAF (95% CI)	OR (95% CI)	*P*_add_

*ACADS*	NGT	4,824 (2,239/2,585)	2,872 (59.5)	1,673 (34.7)	279 (5.8)	23.1 (22.3-24.0)	1.07 (0.96-1.18)	0.21
rs2014355	T2D	3,489 (2,063/1,426)	2,030 (58.2)	1,243 (35.6)	216 (6.2)	24.0 (23.0-25.0)		

*ACADM*	NGT	4,841 (2,237/2,604)	2,353 (48.6)	2,053 (42.4)	435 (9.0)	30.2 (29.3-31.1)	1.01 (0.92-1.11)	0.80
rs11161510	T2D	3,503 (2,083/1,420)	1,686 (48.1)	1,486 (42.4)	331 (9.4)	30.7 (29.6-31.8)		

Data are unadjusted means ± SD or medians (interquartile range). Values of serum insulin/C-peptide and values derived from serum insulin variables were logarithmically transformed before statistical analysis, and their effect sizes are presented as the increase/decrease in percent. All analyses were made using an additive genetic model, adjusting for age, sex, and BMI. The bottom part of the table include number of individuals divided into genotype groups (% in each group), and frequencies of the minor allele (MAF) in percentages. Logistic regression was used to compare allele frequencies (*P*_*add*_*)*. The odds ratios (OR) and the 95% confidence interval (CI) are given for comparison of allele frequency. NGT: Glucose-tolerant individuals, T2D: type 2 diabetic patients. *ACADS *rs2014355 genotypes: WT, homozygous major T-allele carriers; HE, heterozygous; HO, homozygous minor C-allele carriers. *ACADM *rs11161510 genotypes: WT, homozygous major C-allele carriers; HE, heterozygous; HO, homozygous minor T-allele carriers.

**Table 2:** Fasting serum lipid levels of successfully genotyped normoglycemic and hyperglycemic Danes from the Inter99 cohort stratified according to ACADS rs2014355 genotype

	WT	HE	HO	β (95% CI)	***P***_**add**_
**Normoglycemic**					

*n *(men/women)	2576(1208/1368)	1496(694/802)	252(105/147)		

Age (years)	45.2 ± 7.8	45.4 ± 7.9	44.9 ± 7.9		

	Lipid characteristics (mmol/l)				

Total serum cholesterol	5.4 ± 1.0	5.4 ± 1.0	5.4 ± 1.1	0.001 (-0.046; 0.048)	0.97

HDL-cholesterol	1.5 ± 0.4	1.5 ± 0.4	1.5 ± 0.4	0.002 (-0.016; 0.019)	0.86

LDL-cholesterol	3.5 ± 1.0	3.5 ± 1.0	3.5 ± 1.0	-0.001 (-0.065; 0.063)	0.97

VLDL-cholesterol	0.5 ± 0.3	0.6 ± 0.3	0.5 ± 0.2	0.007 (-0.012; 0.026)	0.46

Serum triglyceride	1.0 (0.7-1.4)	1.0 (0.7-1.4)	1.0 (0.7-1.3)	0.5% (-1.8%; 2.7%)	0.69

**Hyperglycemic**					
*n *(men/women)	804(473/331)	496(306/190)	78(49/29)		

Age (years)	48.8 ± 7.4	49.4 ± 7	47.8 ± 8.1		

	Lipid characteristics (mmol/l)				

Total serum cholesterol	5.8 ± 1.1	5.9 ± 1.2	5.9 ± 1.2	0.09 (-0.01;0.19)	0.08

HDL-cholesterol	1.4 ± 0.4	1.3 ± 0.4	1.3 ± 0.5	-0.036 (-0.067; -0.005)	0.02

LDL-cholesterol	3.7 ± 1.0	3.7 ± 1.0	3.9 ± 1.4	0.06 (-0.07; 0.19)	0.38

VLDL-cholesterol	0.7 ± 0.4	0.7 ± 0.4	0.8 ± 0.4	0.03 (-0.02; 0.08)	0.21

Serum triglyceride	1.4 (0.9-2.0)	1.4 (1.1-2.0)	1.5 (1.1-2.6)	5.2% (0.4%; 10.0%)	0.03

Data are unadjusted means ± SD or medians (interquartile range). Values of serum triglycerides were logarithmically transformed before statistical analysis, and their effect sizes are presented as the increase/decrease in percent. Normoglycemic: glucose-tolerant individuals from the Inter99 cohort. Hyperglycemic: individuals with IFG, IGT or screen-detected T2D from the Inter99 cohort. All analyses were made using an additive genetic model, adjusting for age, sex, and BMI. *ACADS *rs2014355 genotypes: WT, homozygous major T-allele carriers; HE, heterozygous; HO, homozygous minor C-allele carriers.

When investigated in all 5,702 treatment-naïve individuals (NGT, IFG, IGT, and screen-detected diabetics) the C-allele of rs2014355 associated with reduced serum insulin levels 30 minutes after an oral glucose load (β = -2.8% (-5.0%;-0.5%), *P *= 0.02), and reduced AUC for serum insulin of (β = -3.2% (-5.7%;-0.7%), *P *= 0.01). The C-allele associated with increased whole body insulin sensitivity estimated according to ISI_Matsuda _(β = 2.4% (0.3%;4.6%), *P *= 0.03) but not with BIGTT-S_i_, HOMA-IR, or disposition index (data not shown). We observed no significant associations between rs11161510 of *ACADM *and any of the investigated quantitative diabetes-related traits (Table 1).

### Case-control analysis of T2D

Case-control studies of rs2014355 of *ACADS *and rs11161510 of *ACADM *were performed in relation to T2D (Table 1); however, none of the variants were significantly associated with the risk of T2D.

The variants were also tested for association with traits related to obesity (BMI, waist, and waist-hip ratio) in both glucose-tolerant (Additional file 2) and in all treatment-naïve individuals; however, no associations were found.

## Discussion

In the present study, we investigated rs2014355 of *ACADS *and rs11161510 of *ACADM *in relation to indices of insulin release and insulin sensitivity as well as their putative relation to the prevalence of T2D. In glucose-tolerant individuals from the Inter99 cohort carrying the *ACADS *rs2014355 minor C-allele we found significantly decreased glucose stimulated insulin release during an OGTT measured as decreased serum insulin levels at 30 min, decreased incremental AUC for serum insulin, decreased BIGTT-AIR, decreased AUC for insulin/AUC for glucose ratio, as well as significantly increased insulin sensitivity in accordance to ISI_Matsuda_. These findings were reproduced, although to a lower significance level when all treatment-naïve individuals from the Inter99 were examined. In contrast, rs11161510 of *ACADM *did not associate with any of the examined quantitative traits or with diabetes in case-control analysis.

We did not find any consistent associations of rs2014355 of *ACADS *with estimates of insulin sensitivity; thus the C-allele did not associate with BIGTT-S_i _or HOMA-IR but only with ISI_Matsuda. _We have no obvious explanation for this inconsistency. Yet, the C-allele also failed to associate with a HOMA-derived disposition index, which is a measure of β-cell function and therefore we cannot exclude that individuals homozygous for the minor C-allele show decreased insulin release as an adaptive response to a primarily improved insulin sensitivity within this genotype group. This interpretation of data is in line with the other insulin sensitivity indices (HOMA-IR and BIGTT-S_i_) that also show a tendency to higher estimates of insulin sensitivity among C-allele carriers, although not statistically significant.

Both T2D and disorders related to *ACADS *show large heterogeneity in the clinical setting which challenges the search for a clear relationship between the two. Additionally, the large variations in phenotypic traits associated with multiple defects of *ACADS *may imply that specific polymorphisms only have a mild regulatory effect. As Gieger *et al. *report, these variants are not associated with severe physiological abnormalities but instead a moderate phenotypic expression may be common in the population. Furthermore, dehydrogenases of other FA's of varying chain-length may partially compensate for the polymorphism and thereby hide the effect or manifest an altered phenotype. Consequently, other interrelating factors, either of environmental or genetic origin, may be necessary to predispose *ACADS *rs2014355 risk allele carriers to β-cell dysfunction.

Under fasting states or other stressing conditions such as fever, defects of *ACADS *have been linked to hypoglycemia [14,26,27]. These findings may be explained by decreased hepatic gluconeogenesis due to FA accumulation and increased utilization of glucose as a fuel because the defective dehydrogenase will prevent the SCFA to be oxidized. However in non-fasting states when there are abundant sources of carbohydrates and fats available, it could be hypothesized, that polymorphisms in *ACADS *would lead to accumulation of SCFA which instead will be converted to complex lipid signaling molecules and thereby synergize with the glucose to stimulate insulin secretion leading to hyperinsulinemia. In the chronic state this could potentially desensitize the insulin receptors, down-regulate insulin release and finally exhaust the β-cell leading to reduced β-cell function.

Together with the additional effect of the increased circulating and tissue FAs, which due to lipotoxicity may operate to diminish the relatively increased insulin sensitivity, a reduced β-cell function could possibly predispose some individuals to T2D. However, the *ACADS *variant did not associate with increased risk of developing T2D in the present case-control study, which is in concordance with the DIAGRAM meta-analysis (stage 1: 4,549 T2D cases and 5,579 control individuals), where rs2014355 shows no association with T2D (OR 1.02 (0.94-1.12), *P*_additive_= 0.6, under the random effects model) [28]. In our study, we are only able to exclude with 80% likelihood that the OR is >1.10. This point to the fact, that none of the two mentioned studies have sufficient statistical power to detect smaller effects of the variant on the risk of developing T2D. It is recognized that many case-control studies of multifactorial diseases with a complex mode of inheritance, e.g., T2D, often are statistically underpowered in yielding conclusive results, and random spurious positive associations due to multiple testing often cannot be excluded. None of the results presented in this paper can withstand correction for multiple testing. Therefore, whether the associations between the *ACADS *variant and examined phenotypes are chance findings, need further investigation in larger well-powered study samples.

## Conclusion

We provide suggestive evidence that the C-allele of rs2014355 of *ACADS *associates with reduced measures of glucose-stimulated insulin release during an OGTT, indicating that the link to T2D previously suggested for this variant, may be mediated through an impaired β-oxidation of fatty acids [10]. However, in the present study we found no evidence of an increased risk of T2D for the C-allele of rs2014355 of *ACADS*. rs11161510 of *ACADM *did not associate with any of the examined T2D related traits. Further investigation is needed before conclusive remarks can be appointed to the variants in *ACADS *and *ACADM *and their putative involvement in the pathogenesis of diabetes.

## List of abbreviations

AIR: acute insulin response; BIGTT-AIR: BIGTT-acute insulin response; BIGTT-S_i_: BIGTT-insulin sensitivity index; CI: confidence interval; CPT-I: carnitine-palmitoyl-transferase I; FA: fatty acids; FFA: free fatty acids; GSIS: glucose-stimulated insulin secretion; GWAS: genome-wide association study; IGT: impaired glucose tolerance; ISI: insulin sensitivity index; MAF: minor allele frequency; MCAD: medium-chain Coenzyme A dehydrogenase; NAFLD: non-alcoholic fatty liver disease; NGT: normal glucose tolerance; OGTT: oral glucose tolerance test; OR: odds ratio; SCAD: short-chain Coenzyme-A dehydrogenase; SCFA: short-chain fatty acids; TG: triglyceride; T2D: type 2 diabetes.

## Competing interests

K. Banasik, C.H. Sandholt, T. Hansen and O. Pedersen hold employee shares in Novo Nordisk A/S. All other authors declare that there is no competing interest associated with this manuscript.

## Authors' contributions

The concept and idea regarding the epidemiological studies underlying the study populations was conceived by CP, AS, TL, OP, and TH. The collection of study subjects was planned and performed by CP, AS, TL, OP, and TH.

The original hypothesis regarding the study was conceived by KB and MH and approved by OP and TS, TH. Detail planning of analyses and study design was performed by KB and MH and approved by OP and TH. KB, MH, JJ, DW, OP, and TH contributed to the establishment of study population databases specific for this study. Statistical analyses in association studies were performed by KB, MH and JJ. NK, CS, and ÅA helped to draft the manuscript. The first manuscript was written by MH, KB, and JJ with equal contribution and the final draft was finalized by KB, TS, OP and TH. All authors revised the manuscript and contributed to the discussion. The final manuscript was read and approved by all authors.

## Pre-publication history

The pre-publication history for this paper can be accessed here:

http://www.biomedcentral.com/1471-2350/12/4/prepub

## Supplementary Material

Additional file 1 **Characteristics for individuals included in the study stratified according to study group**. A table showing the number of individuals included from each study group and their characteristics (as unadjusted means ± SD) Click here for file

Additional file 2 **Anthropometric characteristics of successfully genotyped Danes from the Inter99 cohort**. A table showing the anthropometric characteristics (as unadjusted means ± SD) of successfully genotyped glucose-tolerant Danes from the Inter99 cohort stratified according to *ACADS *and *ACADM *genotypes, including effect sizes and corresponding p-values Click here for file
