# Supplementary material for: The minor C-allele of rs2014355 in ACADS is associated with reduced insulin release following an oral glucose load
Source: BMC Med Genet. 2011 Jan 6;12:4. doi: 10.1186/1471-2350-12-4 (PMC3022800; doi:10.1186/1471-2350-12-4)
Supplement: Additional file 1 — Characteristics for individuals included in the study stratified according to study group. A table showing the number of individuals included from each study group and their characteristics (as unadjusted means ± SD) [file 1471-2350-12-4-S1.DOC]

**Additional files**

**Additional file 1**

**Title: Characteristics for individuals included in the study stratified according to study group**

**Description: A table showing the number of individuals included from each study group and their characteristics (as unadjusted means ± SD)**

|  | Population-based Inter99 study sample | Type 2 diabetic patients from SDC | Population-based study group from SDC | ADDITION Denmark screening study cohort |
| --- | --- | --- | --- | --- |
| Study group | 1 | 2 | 3 | 4 |
| n  (men/women) | 6,162  (3,070 /3,092) | 1,695  (1,045/650) | 810  (382/428) | 1,609  (914/695) |
| Age (years) | 46.2 ± 7.9 | 62.4 ± 11.3 | 58.1 ± 8.4 | 60.2 ± 6.8 |
| Weight (kg) | 78.3 ± 16.1 | 89.7 ± 18.9 | 75.6 ±14.1 | 90.6 ± 17.4 |
| Height (cm) | 172.3 ± 9.2 | 171.7 ± 9.9 | 169.4 ± 9.1 | 170.5 ± 9.0 |
| BMI kg/m^2^ | 26.3 ± 4.6 | 30.3 ± 5.6 | 26.3 ± 4.1 | 31.1 ± 5.4 |

Data are unadjusted means ± SD.
