# Supplementary material for: The minor C-allele of rs2014355 in ACADS is associated with reduced insulin release following an oral glucose load
Source: BMC Med Genet. 2011 Jan 6;12:4. doi: 10.1186/1471-2350-12-4 (PMC3022800; doi:10.1186/1471-2350-12-4)
Supplement: Additional file 2 — Anthropometric characteristics of successfully genotyped Danes from the Inter99 cohort. A table showing the anthropometric characteristics (as unadjusted means ± SD) of successfully genotyped glucose-tolerant Danes from the Inter99 cohort stratified according to ACADS and ACADM genotypes, including effect sizes and corresponding p-values [file 1471-2350-12-4-S2.DOC]

**Additional file 2**

**Title: Anthropometric characteristics of successfully genotyped Danes from the Inter99 cohort**

**Description: A table showing the anthropometric characteristics (as unadjusted means ± SD) of successfully genotyped glucose-tolerant Danes from the Inter99 cohort stratified according to *ACADS* and *ACADM* genotypes, including effect sizes and corresponding p-values**

| ***ACADS*** | | | | | |
| --- | --- | --- | --- | --- | --- |
| **Glucose-tolerant individuals (*n=*4,324)** | | | | | |
|  | **TT** | **TC** | **CC** | **β (95% CI)** | ***P*_add_** |
| *n* (men/women) | 2,576 (1,208/1,368) | 1,496 (694/802) | 252 (105/147) |  |  |
| Age (years) | 45±8 | 45±8 | 45±8 |  |  |
| BMI (kg/m^2^) | 25.5±4.1 | 25.4±3.9 | 25.7±4 | 0.03 (-0.16;0.23) | 0.74 |
| Waist hip ratio | 0.8±0.1 | 0.8±0.1 | 0.8±0.1 | -0.002 (-0.004;0.001) | 0.21 |
| Waist (cm) | 84.3±12.1 | 83.9±12.4 | 83.9±11.6 | -0.20 (-0.46;0.05) | 0.11 |
| ***ACADM*** | | | | | |
| **Glucose-tolerant individuals (*n*=4,337)** | | | | | |
|  | **CC** | **CT** | **TT** | **β (95% CI)** | ***P*_add_** |
| *n* (men/women) | 2,121(991/1,130) | 1,821(825/996) | 395(186/209) |  |  |
| Age (years) | 45±8 | 45±8 | 46±8 |  |  |
| BMI (kg/m^2^) | 25.5±4.0 | 25.6±4.2 | 25.4±3.8 | 0.03 (-0.16;0.21) | 0.79 |
| Waist hip ratio | 0.8±0.1 | 0.8±0.1 | 0.8±0.1 | -0.002 (-0.004;0.001) | 0.18 |
| Waist (cm) | 84.2±12.1 | 84.1±12.5 | 84.1±11.5 | -0.07 (-0.31;0.16) | 0.55 |

Data are unadjusted means ± SD. All analyses were made using an additive genetic model, adjusting for age, sex, and BMI.
